# An algorithm for the EEG frequency architecture of consciousness and brain body coupling

**DOI:** 10.3389/fnhum.2013.00766

**Published:** 2013-11-12

**Authors:** Wolfgang Klimesch

**Affiliations:** Department of Physiological Psychology, University of SalzburgSalzburg, Austria

**Keywords:** EEG, oscillations, brain body coupling, scale free EEG algorithm, harmonic oscillations, golden mean

## The frequency architecture of the conscious human brain

**Figure 1:**
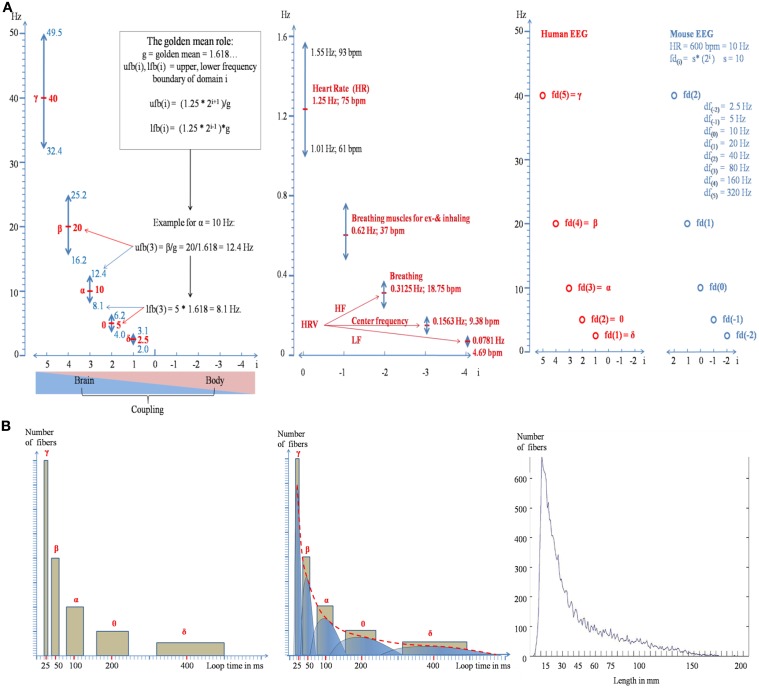
**Illustration of the doubling-halving algorithm as described by formula (2a)**. **(A)** The distribution of the frequency domains, together with their bandwidths is shown in the left panel for brain oscillations and in the middle panel for body oscillations. The frequency boundaries are calculated according to the “golden mean role”: The upper frequency boundary of domain i is that frequency which is maximally separated from domain *i* + 1 and the lower boundary is that frequency which is maximally separated from domain *i* − 1 (see the inset in the left panel for an example). The predicted frequency architecture for a mouse with a heart rate of 600 bpm (=10 Hz) is shown in the right panel. Note that the values for the center frequencies are the same as for humans but the relation to the index of a domain is changed. **(B)** Formula (2) can be used to predict the distribution of long-range white matter connectivity. The areas of the yellow rectangles in the left and middle panel represent the percentage of bundles for a frequency domain. Note that the area of each rectangle is constant and that the two sides of the rectangles change according to the doubling-halving algorithm of formula (2). The empirical distribution is shown in the right panel (data are from Hagmann et al. (2008), provided by Olaf Sporns).

The EEG is traditionally categorized into a handful of different frequency bands (δ, θ, α, β, γ; c.f. Schomer and Lopes da Silva, 2011). This implies that EEG frequencies do not represent an unstructured continuum. But what could be the reasons for that? One obvious reason is that frequency bands such as e.g., θ and α exhibit a clear task and event related behavior (Klimesch, 1999, 2012; Buzsaki, 2006). But here the emphasis is on a formal aspect, which is to avoid unwanted “spurious” phase synchronization. If the numerical ratio between two frequencies (*f*_1_, *f*_2_; *f*_1_ < *f*_2_) is harmonic (*f*_2_ = *I* * *f*_1_; *I* = integer), the excitatory phases of the two frequencies can meet and synchronize according to a strict and regular pattern. This is of great advantage when phase coupling between frequencies is an important aspect of neuronal communication. If the ratio differs from a harmonic, spurious (unwanted) phase synchronization will appear in an uncontrolled way. Pletzer et al. (2010) have shown mathematically that the golden mean (*g* = 1.618 ….) is the best possible ratio to avoid spurious phase synchronization (see also Roopun et al., 2008). These aspects of phase synchronization can be summarized by two assumptions. (a) The center frequency of each EEG band is harmonically related to those of neighboring bands. A good estimate for δ, θ, α, β and γ is 2.5, 5, 10, 20, and 40 Hz. (b) The width of a band is defined on the basis of the “golden mean role” (Klimesch, 2012; for an illustration, see Figure 1A left panel) to guarantee minimal interference between bands. EEG center frequencies which have these properties are termed frequency domains in the following.

When considering the numerical relation between δ, θ, α, γ as described by assumption (a) it can easily be recognized that each center frequency is twice as high than its (lower) neighbor. An exponential function with base 2 defines that property.

(1) $$I(i)=2^{i}i=0,1,2,3,\ldots .$$

     I =1,2,4,8,….

Variable *I*(*i*) describes the numerical relation between δ, θ, α, γ as a series of harmonic frequencies that increase (or decrease) with i. As an example, we can multiply the frequency of δ by *I*(*i*) to calculate the entire series of harmonic frequencies: 2.5 * *I*_(*i* = 0)_ = 2.5 Hz; 2.5 * *I*_(*i* = 1)_ = 5 Hz; 2.5 * *I*_(*i* = 2)_ = 10 Hz; 2.5 * *I*_(*i* = 3)_ = 20 Hz; 2.5 * *I*_(*i* = 4)_ = 40 Hz. This exponential function represents a scale free law of doubling and halving any frequency to obtain neighboring harmonics. It generates a “chain” of harmonics with a “dual” structure.

It is well known that individual EEG frequencies depend on basic biological factors such as age, gender and possibly brain size (Nunez et al., 1978; Köpruner et al., 1984; Valdés-Hernández et al., 2010; Nunez, 2011). Thus, let us introduce a scaling factors that “scales” the numerical values of all frequency domains *fd*(*i*):

(2) $$fd(i)=s*2^{i}$$

An estimate for s can be determined by substituting the respective values in formula (2). When considering δ the first frequency in the harmonic series, we have *i* = 1 and *fd*(*i*) = 2.5 Hz. When solving for s we obtain 1.25. It is now possible to suggest a simple definition of a “basic frequency,” as that frequency with a value of *i* = 0: *fd*(*i* = 0) = 1.25 * 2^0^ = 1.25 Hz. The interesting point here is that *f*_(0)_ *is* the scaling factor:

(2a) $$fd(i)=f_{\left(0\right)}*2^{i}=1.25*2^{i}$$

Is there a specific biological meaning of *f*_(0)_? The frequency of 1.25 Hz equals 75 beats per minute (bpm) which is very close to the average heart rate (HR) of young adults. This suggests that HR—which is known to vary with body size, age and sex—is the basic frequency and the scaling factor for all other frequency domains.

It is interesting to see that formula (2a) does not only give a faithful description of the numerical values of traditional EEG frequencies and their bandwidths but also of those of body oscillations (for *i* < 1). As illustrated in the middle panel of Figure 1A, these frequencies are close to the brainstem oscillations that trigger inhaling and exhaling (resulting in two excitatory events for one breathing cycle), breathing frequency, and heart rate variability (HRV).

## Predictions and implications

### White matter connectivity

There is a traditional belief that fast frequencies are associated with smaller networks and slow frequencies with larger networks (cf. Von Stein and Sarnthein, 2000). Here it will be shown that formula (2) can be used to predict the architecture of white matter connectivity which has meanwhile been well-described by DTI and DSI tractographical methods (e.g., Hagmann et al., 2008). We proceed from the following considerations. Let us assume that EEG frequencies reflect activity of “long-range” cortico-cortical networks (Varela et al., 2001) that are connected by myelinated axons. Let us further assume that the frequency of a domain is related to the size of a network which is characterized by a preferred length of reciprocal connections. When we assume that loop time—the conduction time for traversing a reciprocal connection—is equal to the period of a frequency domain (see the theory of resonant, self-organizing phase locked loops by Miller, 1991), we can predict the frequency histogram of loop times for each domain. The “doubling-halving” nature of our algorithm predicts that loop time of a given domain i is twice as long than that of *i* − 1 but half of that of *i* + 1. The scale free nature of the algorithm suggests that there is no “typical” domain. Thus, we assume that the network of each domain consists—approximately—of an equal number of connections. This means that the percentage of fibers belonging to a certain domain is a comparatively constant number. If we would assume that δ, θ, α, β, γ are associated with long range white matter connections, each frequency domain would be associated with about 20% of all white matter fibers, as is illustrated in the left panel of Figure 1B. An obvious problem with this distribution is that we see “gaps” in loop length. We can overcome this problem by assuming that loop time can stay constant when longer fibers are stronger myelinated than shorter fibers (conduction velocity is positively associated with myelin shield thickness; e.g., Rushton, 1951; Goldman and Albus, 1968; Sabah, 2000). Thus, a differential and selective myelination allows us to predict that loop times do not overlap between different frequency domains, although they have (different) fibers with the same length. This idea is illustrated in the middle panel of Figure 1B, where each bar is replaced by an asymmetric inverted u shape distribution, with the right part representing strongly and the left part weakly myelinated fibers. The resulting distribution is represented by the red dotted line. Several findings are consistent with this prediction. First, there is some evidence that myelin thickness is positively correlated with fiber length (Chen et al., 1992; Hursh, 1939) too keep conduction latency constant irrespective of fiber length (Salami et al., 2003). Second, and most importantly, the predicted distribution is strikingly similar to the empirically observed distribution of fiber lengths as shown in the right panel of Figure 1B (data are from Hagmann et al., 2008, provided by Olaf Sporns). It should also be noted that the predicted shape of the distribution does not agree with the concept of “random connectivity.” When the cortex is modeled as the surface of a sphere, the resulting distribution of connections between all points of the surface exhibits an inverted u-shape distribution around the radius as mean.

### Global harmonic synchronization and conscious cognition

The chain of harmonic frequency domains as described by formula (2) may be considered a coordinate system for global synchronization, which most likely is typical for conscious cognition. This is well in line with the idea that consciousness is associated with coherent global and long-range brain processes (Bressler and Menon, 2010; Dehaene and Changeux, 2011). Three groups of empirical data are also in support of this view, the task dependent emergence of between frequency phase coupling (e.g., Palva et al., 2005), the observation that ERP's can be described by a superposition of transiently phase coupled frequencies (Klimesch et al., 2007) and—most importantly—that a change in the state of consciousness from active cognition to drowsiness and slow wave sleep (SWS) is accompanied by a dramatic change in the frequency architecture. It is characterized by a decoupling between those frequency domains that are described by formula (2a) and the emergence of frequencies (slow waves and spindles) that do not play a role during conscious cognition. SWS may be characterized by a loss of phase coupling and the emergence of phase to amplitude envelope coupling between slow waves and spindles (e.g., Steriade, 2006).

### Heart rate as scaling factor

An important and surprising implication of formula (2a) is that HR and brain oscillations on the one hand and HR and body oscillations on the other hand can be harmonically coupled. One obvious question that arises is, whether changes in HR may lead to a direct, concomitant change in the frequency of brain (and body) oscillations. Brain oscillations may change slightly (e.g., α may exhibit a fatigue related decrease of about 1or 2 Hz) but never to an extent as HR is capable. Thus, in most cases a direct coupling with brain and body oscillations will not be possible. Two aspects are important here. One refers to a state of decoupling between brain oscillations and HR if the change in HR is very pronounced, such as during heavy exercise. The other aspect refers to an adaptive change in HR that may indeed allow a direct but short lasting, transient, coupling with brain oscillations even in cases where HR is tonically increased or decreased. As an example, if HR is increased to 90 bpm (1.5 Hz; period of 667 ms) a transient decrease to 75 bpm (i.e., an increase in the period of 800 ms for a few heart beats) or increase to 150 bpm (a decrease in the period to 400 ms for a few heart beats) would still allow for a transient task-related harmonic coupling with brain oscillations. Such an adaptive mechanism could be responsible for the generation of HRV. It is interesting to note that HR may also operate to “reset” brain activity as the existence of heartbeat evoked potentials suggest (Dirlich et al., 1998).

## Conclusions: brain and body as coupled oscillators.

Our algorithm can be considered a coordinate system for the coupling of brain and body oscillations. Brain body interactions may, thus, be described as complex system that couples and decouples (Buchman, 2002) on the basis of a specific harmonic frequency structure. As a scale free law it probably underlies all animal species. The fact that HR exhibits a tremendous between species variation (about 600 bpm for rats and 20 bpm for elephants) means—according to formula (2a)—that the physiological function of the domains change although the absolute frequency values may change little or remain even identical [if the animal *fd*_(0)_ obeys the doubling-halving algorithm relative to human HR] as is illustrated in the right panel of Figure 1.

In a mathematic sense formula (2) represents a binary system. Since the emergence of information theory it became clear that any kind of information can be encoded on the basis of binary units (e.g., Strogatz, 2012, p. 40). One may speculate that this algorithm represents a basic physical law of information encoding that requires the least amount of energy. This view is based on the fact that the scaling factor s has far reaching consequences from HR to body and brain size to metabolic processes. Another aspect is its fractal property because the doubling-halving relationship repeats over all different scales. For future research, the establishment of a large normative data base for brain and body oscillations would be helpful to clarify the questions raised here.
